# CRP/Albumin Ratio and Glasgow Prognostic Score Provide Prognostic Information in Myelofibrosis Independently of MIPSS70—A Retrospective Study

**DOI:** 10.3390/cancers15051479

**Published:** 2023-02-25

**Authors:** Nora-Medea Messerich, Narasimha Rao Uda, Thomas Volken, Sergio Cogliatti, Thomas Lehmann, Andreas Holbro, Rudolf Benz, Lukas Graf, Vikas Gupta, Wolfram Jochum, Izadora Demmer, Tata Nageswara Rao, Tobias Silzle

**Affiliations:** 1 Department of Intensive Care, Cantonal Hospital St. Gallen, 9007 St. Gallen, Switzerland; 2 Laboratory of Stem Cells and Cancer Biology, Department of Oncology and Hematology, Medical Research Center, Cantonal Hospital St. Gallen, 9007 St. Gallen, Switzerland; 3 ZHAW School of Health Sciences, Institute of Public Health, 8400 Winterthur, Switzerland; 4 Institute of Pathology, Cantonal Hospital St. Gallen, 9007 St. Gallen, Switzerland; 5 Clinic for Medical Oncology and Hematology, Cantonal Hospital St. Gallen, 9007 St. Gallen, Switzerland; 6 Division of Hematology, University Hospital of Basel, University of Basel, 4001 Basel, Switzerland; 7 Division of Hematology and Oncology, Spital Thurgau AG, 8569 Muensterlingen, Switzerland; 8 Centre for Laboratory Medicine, 9001 St. Gallen, Switzerland; 9 Princess Margaret Cancer Center, University of Toronto, Toronto, ON M5S 1A1, Canada; 10 Institute for Pharmacology, University of Bern, 3012 Bern, Switzerland

**Keywords:** myelofibrosis, C-reactive protein, albumin, CRP/albumin ratio, Glasgow Prognostic Score, MIPSS70, prognostication

## Abstract

**Simple Summary:**

To assess prognosis in myelofibrosis (MF), age and degree of anemia and leukocytosis are taken into account together with the presence of blasts in the peripheral blood and constitutional symptoms (fever, night sweats, weight loss). The latter are signs of systemic inflammation, which plays a pivotal role in MF pathophysiology. Considering information about genetic changes can refine prognostication. The goal of our retrospective study was to assess the prognostic impact of two laboratory markers of inflammation that are readily available in clinical routine at low costs: C-reactive protein (CRP) and albumin. We found a significant prognostic impact of both parameters either alone or combined within the CRP/albumin ratio or the Glasgow Prognostic Score, which was independent of the Mutation-Enhanced International Prognostic Scoring System (MIPSS)-70. Therefore, assessing CRP and albumin helps to identify a vulnerable population of MF patients, which eludes current prognostic models, even if the presence of high-risk mutations is considered.

**Abstract:**

In myelofibrosis, the C-reactive protein (CRP)/albumin ratio (CAR) and the Glasgow Prognostic Score (GPS) add prognostic information independently of the Dynamic International Prognostic Scoring System (DIPSS). Their prognostic impact, if molecular aberrations are considered, is currently unknown. We performed a retrospective chart review of 108 MF patients (prefibrotic MF n = 30; primary MF n = 56; secondary MF n = 22; median follow-up 42 months). In MF, both a CAR > 0.347 and a GPS > 0 were associated with a shorter median overall survival (21 [95% CI 0–62] vs. 80 months [95% CI 57–103], *p* < 0.001 and 32 [95% CI 1–63] vs. 89 months [95% CI 65–113], *p* < 0.001). Both parameters retained their prognostic value after inclusion into a bivariate Cox regression model together with the dichotomized Mutation-Enhanced International Prognostic Scoring System (MIPSS)-70: CAR > 0.374 HR 3.53 [95% CI 1.36–9.17], *p* = 0.0095 and GPS > 0 HR 4.63 [95% CI 1.76–12.1], *p* = 0.0019. An analysis of serum samples from an independent cohort revealed a correlation of CRP with levels of interleukin-1β and albumin with TNF-α, and demonstrated that CRP was correlated to the variant allele frequency of the driver mutation, but not albumin. Albumin and CRP as parameters readily available in clinical routine at low costs deserve further evaluation as prognostic markers in MF, ideally by analyzing data from prospective and multi-institutional registries. Since both albumin and CRP levels reflect different aspects of MF-associated inflammation and metabolic changes, our study further highlights that combining both parameters seems potentially useful to improve prognostication in MF.

## 1. Introduction

Both primary and secondary myelofibrosis (PMF/SMF) are caused by a complex interplay of (epi)genetic alterations in hematopoietic stem cells and inflammatory changes, which affect hematopoiesis and impact patient survival [1,2]. 

The Dynamic International Prognostic Scoring System (DIPSS) as a standard tool for prognostication considers age, anemia, leukocytosis, peripheral blast counts and constitutional symptoms [3]. It can be refined by incorporating information about cytogenetic aberrations, mutational profile or both [4]. In addition to these complex and expensive parameters, some routine laboratory markers add prognostic information, such as C-reactive protein (CRP) and albumin. Elevated CRP levels have been associated with several adverse disease features and a shorter leukemia-free survival [5,6], and albumin has been consistently shown to add additional prognostic information independently of DIPSS and several DIPSS-based prognostic scoring systems [7,8,9]. Furthermore, indices combining CRP and albumin such as the CRP/albumin ratio (CAR) [10] or the Glasgow Prognostic Score (GPS) [11] provide DIPSS-independent prognostic information in MF.

With regard to both CAR and GPS, it remains elusive as to whether they still add prognostic value if the molecular risk profile is considered. We therefore examined the prognostic impact of CAR and GPS in relation to the Mutation-Enhanced International Prognostic Scoring System (MIPSS)70, which includes the mutational profile without needing conventional metaphase cytogenetics [12].

## 2. Patient Population and Methods

We performed a retrospective chart review of patients diagnosed with MF at the Cantonal Hospital St. Gallen between 2000 and 2020 (Cohort A).

One hundred and eight patients were identified (47 female and 61 male, median age 72; pre-fibrotic MF: 30/108 (28%), PMF 56/108 (52%) and SMF 22/108 (20%)), and clinical and laboratory data were collected at the time of diagnosis and before commencement of treatment. All of the cases were reviewed individually, to ensure correct classification according to WHO2016 [13]. If the diagnostic work-up did not include next-generation sequencing (NGS), we performed mutational profiling using material from the diagnostic samples (see the Supplementary Materials “Supplementary Methods”). Detailed patient characteristics of the cases with MF in cohort A (PMF and SMF) are shown in Table 1.

The CAR was calculated by dividing the CRP concentration (mg/L) by the albumin concentration (g/L). The GPS was determined according to [14] (GPS 0: albumin ≥ 35 g/L and CRP ≤ 10 mg/L; GPS 1: either albumin < 35 g/L or CRP > 10 mg/L; GPS 2: both albumin < 35 g/L and CRP > 10 mg/L).

For CRP, we used the upper limit of normal from our local laboratory for dichotomization (≤/>8 mg/L), and for albumin, the median of our population was used (</≥40 g/L). For the CAR, we used a cut-off of </≥0.204, as proposed by [10] and a CAR of </≥0.374, representing the fourth quartile of our cohort. The methods applied for the statistical analysis are described in detail in the Supplementary Materials.

Plasma probes from an independent Canadian cohort (Cohort B) of 64 MPN patients (MF n = 28, PV n = 18, ET n = 18; Supplementary Table S1) and healthy controls (n = 16) were available to assess the correlation of high-sensitivity (hs)CRP and albumin levels with pro-inflammatory cytokines, which were measured as described in detail in the Supplementary Materials.

## 3. Results

### 3.1. Levels of CRP and Albumin, the CAR in Different MF Subgroups and Their Association with Disease Characteristics

Within Cohort A, we found higher levels of conventional CRP in patients with MF (PMF: n = 56, median 5 mg/L, [IQR 2–18], SMF: n = 22, median 5 mg/L [IQR 3–9]) compared to pre-fibrotic MF (n = 30, median 1 mg/L, [IQR 1–8], *p* = 0.034). With regard to the albumin concentration, we found no difference (PMF median 40.5 g/L [IQR 37–42.6], SMF median 39 g/L [IQR 36.4–42.7], pre-fibrotic MF median 42 g/L [IQR 38–43.6], *p* = 0.253).

In MF, a CRP-elevation > 8 mg/L was associated with lower levels of hemoglobin and platelets, a higher percentage of peripheral blasts, higher LDH-levels, transfusion-dependency and the presence of constitutional symptoms, whereas levels of albumin < 40 g/L were associated only with the degree of anemia and with a lower body mass index (BMI), as shown in Table 1. An additional comparison of disease characteristics following the cut-offs used within the GPS (CRP ≤/> 10 mg/L and albumin </≥ 35 g/L) is provided in Supplementary Table S2. 

There was no difference in CRP, albumin and the CAR between JAK2-mutated cases and CALR-mutated cases. With regard to JAK2-V617F variant allele frequency (VAF), we observed a significantly higher CAR in patients with a VAF > 50% (median 0.243 vs. 0.095, *p* = 0.035) and a trend towards higher CRP values (median 7.5 vs. 4.5 mg/L, *p* = 0.071). No difference was noted for albumin (median 39 vs. 38 g/L, *p* = 0.158). Patients with high-risk mutations according to MIPSS70 showed a tendency towards a higher CAR (median 0.579 vs. 0.115, *p* = 0.051) but did not differ significantly with regard to the single parameters. Further details are shown in Supplementary Table S3.

MIPSS70 was available for 59/78 patients (76%): intermediate risk 43/59 (72.9%), high risk 14/59 (23.7%) and low-risk 2/59 (3.4%). Overall survival (OS) different significantly among these groups (Supplementary Figure S1).

Compared to the MIPSS70-intermediate patients, the MIPSS70-high-risk patients had significantly higher CRP levels (median 14 mg/L [IQR 5–30] vs. 5 mg/L [IQR 1–10], *p* = 0.012), but not lower albumin levels (median 38 vs. 39 g/L, *p* = 0.224). Accordingly, the CAR was higher in MIPSS70-intermediate patients (median 0.504 [95% CI 0.95–0.739] vs. 0.116 [95% CI 0.026–0.255,], *p* = 0.025). Given their low number, we did not include the MIPSS70-low risk group in this analysis. 

### 3.2. Prognostic Impact of CRP, Albumin and Derived Indices (CAR and GPS) in MF

The probability of death rose continuously with lower albumin levels even in the range determined as normal (OR = 0.85, 95% CI 0.73–0.99; *p* = 0.043, Supplementary Figure S2), and an albumin concentration below the population median was associated with a significantly shorter survival (albumin </≥ 40 g/L, median OS 50 [95% CI 38–62] vs. 101 [95% CI 51–151] months, *p* = 0.026). CRP > 8 mg/L (n = 24) was associated with shorter survival compared to CRP within the normal limits (≤8 mg/L, n = 47): median OS 44 [95% CI 0–89] vs. 89 [95% CI 56–122] months, *p* < 0.001.

**Figure 1:**
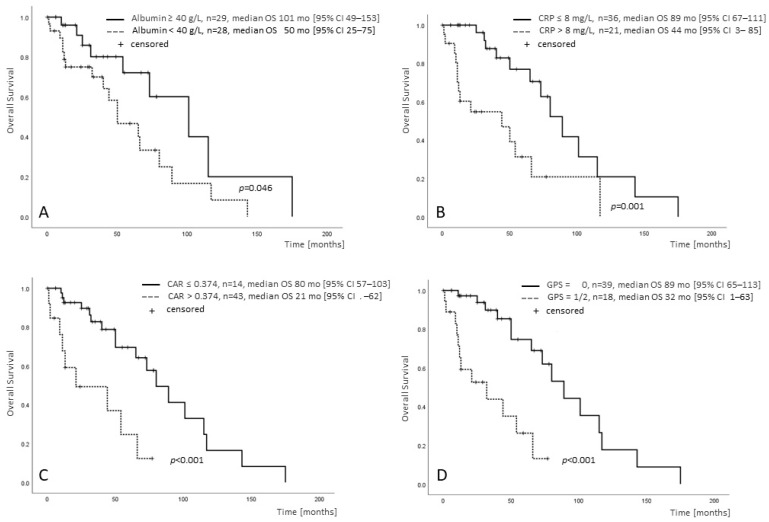
Prognostic significance of CRP, albumin and the CRP/albumin ratio in MF as assessed by univariate analyses. Survival of patients with MF, for whom both albumin and CRP were available (n = 57), stratified by albumin </≥ 40 g/L (**A**), CRP ≤/> 8 mg/L (**B**), CRP/albumin ratio ≤/> 0.374 (**C**) and GPS </> 0 (**D**).

Correspondingly, a higher CAR was associated with inferior survival (median OS CAR ≤/> 0.204: 89 [95% CI 67–111] vs. 44 [95% CI 3–85] months, *p* = 0.001; and CAR ≤/> 0.374: 80 [95% CI 57–103] vs. 21 [95% CI 0–62] months, *p* < 0.001). Similar results were obtained for patients with a GPS of 1 or 2 (n = 18) compared to patients with a GPS of 0 (n = 39): median OS 32 [95% CI 1–63] vs. 89 [95% CI 65–113] months, *p* < 0.001. Kaplan–Meier curves for the patients for whom both CRP and albumin were available (n = 57) are shown in Figure 1A–D.

For all of the factors, a higher HR for mortality was observed in univariate Cox regression models (Table 2). Given the low number of MIPSS70-low-risk patients (n = 2), we dichotomized the cohort into a “MIPSS70dich^low/intermediate^” risk group (n = 45) and a “MIPSSdich^high^” risk group (n = 14) for analyses in bivariate models. Here, CRP > 8 mg/L, albumin < 40 g/L, and both a CAR > 0.374 and a GPS > 0 retained their prognostic value together with MIPSS70dich, whereas a CAR > 0.204 did not (Table 2). In a separate analysis considering only the PMF patients (n = 35) and applying the same threshold for CAR (>0.374) and GPS (>0), the results remained significant, albeit with large 95% confidence intervals (Table 3). Of note, for SMF, the very low number of cases (n = 12) for whom both CRP and albumin were available precluded a separate analysis. 

**Figure 2:**
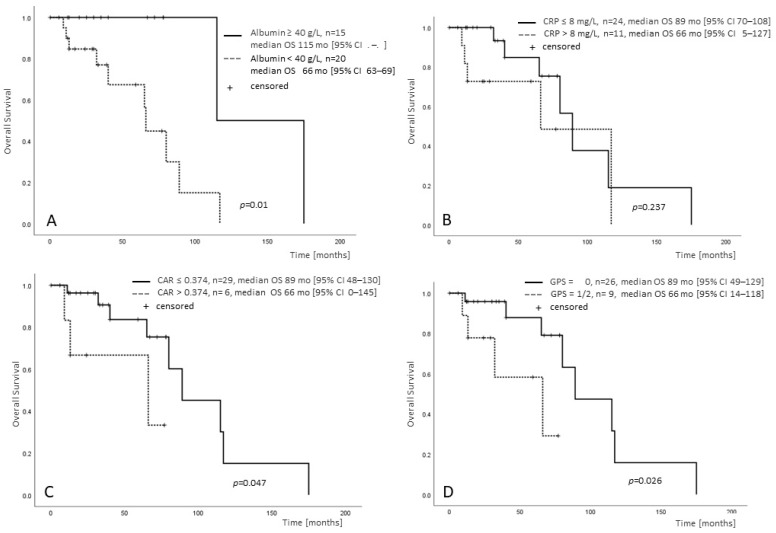
Albumin and CRP provide MIPSS70-independent prognostic information in MF. Survival of MIPSS70-intermediate-risk patients, for whom both albumin and CRP were available (n = 35), stratified by albumin </≥ 40 g/L (**A**), CRP ≤/> 8 mg/L (**B**), CRP/albumin ratio ≤/> 0.374 (**C**) and GPS </> 0 (**D**).

For MIPSS70-intermediate patients with both CRP and albumin available (n = 35), OS was significantly shorter for albumin < 40 g/L, CAR > 0.374 and GPS > 0, whereas CRP ≤/> 8 mg/L was not associated with an adverse prognosis (Figure 2A–D).

### 3.3. Association of Levels of CRP and Albumin with Inflammatory Cytokines

Analysis of cohort B showed higher levels of hsCRP (median 10.07 vs. 7.02 mg/L; *p* < 0.0004) and lower levels of albumin (median 31.4 vs. 25.87 g/L; *p* = 0.0012) in MF versus MPN without fibrosis and/or the healthy controls. The VAF of the driver mutation was correlated only to levels of hsCRP (*p* = 0.008) (Supplementary Figures S3 and S4). The levels of interleukin-1β, interferon-γ, CCL17, I-TAC and ENA-78/CXCL-5 correlated positively with hsCRP, while no significant correlation was observed for IL-6, TNFα, IFNα, IL-8, IL-18, IL-10, IL-33, IL-17a, IL-23 and MCP-1 (Supplementary Figures S5 and S6). Albumin levels were inversely correlated to TNFα and MCP-1 (Supplementary Figures S7 and S8).

## 4. Discussion

CRP and albumin resemble surrogate markers for the extent of inflammation, a key element of MPN pathophysiology ([1,15]). Higher CRP levels are known to be associated with shortened leukemia-free and overall survival in univariate analyses [5,6], whereas for albumin, a prognostic value independent of several DIPPS-based scoring systems has been described previously [7,8,9]. As expected, we therefore found a significant impact of both parameters on survival in our cohort. 

Levels of CRP were more closely related to the established adverse features of MF, which are in part or indirectly taken into account by current models, e.g., peripheral blasts, more severe anemia and/or transfusion-dependency or thrombocytopenia < 100 × 10^9^/L, whereas only lower albumin levels were associated with a lower BMI as a measure of MF-induced cachexia. In addition, both factors were associated with levels of different cytokines, namely CRP with interleukin-1β, a driver of MF pathogenesis [16,17], and albumin with TNF-α, a key mediator of cachexia [18]. This implies that CRP and albumin probably reflect different aspects of MF pathophysiology. It is therefore of interest to combine them in the CAR or the GPS. For both parameters, a DIPSS-independent prognostic value has already been described in MF [10,11]. A recent report on acute myeloid leukemia patients not eligible for stem-cell transplantation illustrates that a combined assessment of CRP and albumin is of interest in myeloid malignancies in general [19]. 

We found a MIPSS70-independent prognostic value for both a CAR > 0.347 and GPS > 0. Hence, both parameters add prognostic information, even in the context of a molecular prognostic score. However, the relevant cut-off for the CAR used within our MIPSS70-based model was higher than that published for DIPSS-based prognostication [10]. This might be due to different composition of the patient populations, different access to potentially disease-modifying drugs such as ruxolitinib or the influence of age, which is part of the DIPSS but not the MIPSS70. Further studies are needed to define the optimal cut-off of the CAR to be used in the context of the single different scoring systems and/or to decide whether CAR or the GPS provides better prognostic information. 

Malnutrition and/or activation of catabolic pathways leading to hypoalbuminemia are probably not sufficient to explain the prognostic impact of albumin, since levels still in the lower range of normal represent an adverse risk factor not only in our cohort, but also according to all of the reports currently available on the prognostic role of albumin in MF [7,8,9]. Several pleiotropic effects of albumin have been described [20]. Amongst others, it represents the main anti-oxidant in the extracellular space [21], and higher levels could be associated with an increased capability to counteract ROS-mediated inflammation, which is linked to disease progression [22] in MF. This would indicate a vicious cycle, if inflammation has reached a point where albumin synthesis is limited. However, this hypothesis warrants confirmation in further studies.

Considering albumin and CRP in clinical practice evidently helps to identify a more vulnerable population of MF patients who elude current prognostic models and could benefit from multimodal interventions. Both markers are associated with cardiovascular risk [23,24]; therefore, modifiable risk factors should be aggressively managed in MF patients with low albumin and elevated CRP levels and/or a higher CAR. The JAK2 inhibitor ruxolitinib controls not only constitutional symptoms and splenomegaly, but also lowers CRP levels and increases albumin concentration [25]. This may justify its use even in low-risk patients harboring one of the risk factors based on CRP and albumin, especially if splenomegaly is already present. Non-pharmacological interventions, such as physical exercise and nutritional interventions, can positively affect both parameters [26,27]. In this context, the Mediterranean Diet is currently under investigation in MF [28].

As this was a monocentric and retrospective study, the interpretation of our observations is subject to several limitations. Apart from a potential selection bias, the limited number of patients is most relevant, since it precludes defining the cut-off of the CAR that is best suited for prognostication or adjusting for possibly confounding factors such as age and treatment with disease-modifying drugs such as ruxolitinib. Due to the low number of patients, we had to combine cases of primary and secondary MF. Whether prognostic scores established for PMF are of value for patients with SMF is still a matter of debate [29,30], and the Myelofibrosis Secondary to PV and ET-Prognostic Model (MYSEC-PM) was developed especially for this population [31]. However, the MYSEC-PM does not consider the presence and type of additional non-driver mutations; hence, the MIPSS70 represents one of the currently suggested tools for prognostication in both PMF and SMF, if the mutational profile has to be considered [32]. A further limitation is the fact that conventional metaphase cytogenetics were available only for a minority of patients, precluding the assessment of the factors studied in the context of scoring systems, which consider chromosomal aberrations in addition to the mutational profile, such as the MIPSS70+ Version 2.0 [33].

## 5. Conclusions

Our data have shown for the first time that CAR and GPS add prognostic information independently of the MIPSS70-based molecular risk profile in MF. Albumin and CRP are easily available in clinical routine at low cost and represent potential biomarkers to faithfully identify a more vulnerable population of MF patients not identified by current prognostic model systems. Moreover, since CRP and albumin probably reflect different aspects of MF pathophysiology, including inflammation and metabolic aspects, combining both parameters seems particularly useful for MF prognostication. However, further studies involving multi-center registries with larger cohorts are necessary to validate the prognostic impact of albumin and CRP within the context of prognostic scoring systems considering both cytogenetics and the mutational status. In addition, it remains to be determined as to whether improving levels of CRP and albumin during therapy are associated with a better prognosis. Despite all limitations, our observations fit well into the emerging data and support the prognostic role of albumin and CRP in MF.

## Figures and Tables

**Table 1:** Characteristics of patients with MF from in Cohort A including PMF (56/78) and MF post ET/PV (22/78). Data are shown for the whole population and according to the levels of CRP and albumin.

	Whole Population	CRP ≤8 mg/L	CRP >8 mg/L	*p*	Albumin ≥ 40 g/L	Albumin < 40 g/L	*p*
n	78	47	24		31	30	
Age [years], median, (IQR)	72 (60–78)	70 (60–77)	76 (63–80)	0.068	69 (60–77)	76 (67–79)	0.075
Female n, (%)	37 (46.2)	24/47 (51)	10/24 (41.7)	0.616	14/31 (45.2)	16/30 (53.3)	0.612
Bone marrow fibrosis grade 2, n (%)	54/78 (70)	37/47 (78.7)	14/24 (58.3)		25/31 (80.6%)	20/30 (67)	
Bone marrow fibrosis grade 3, n (%)	24/78 (30)	10/47 (21.3)	10/24 (41.7)	0.096	6/31 (19.4)	10/30 (33)	0.255
Hemoglobin [g/L], median (IQR)	107 (88–122)	117 (102–131)	86 (77–103	<0.001	115 (103–130)	96 (80–113)	0.003
Platelet count (×10^9^/L) median (IQR)	410 (197–663)	550 (340–773)	255 (110–442)	0.001	541 (200–770)	391 (231–576)	0.329
Leukocytes (×10^9^/L), median (IQR)	8.9 (6.0–15.6)	9.5 (6.8–16)	8.7 (5.5–20)	0.551	9.4 (6.8–16)	10 (6.3–21)	0.751
Neutrophils (×10^9^/L), median (IQR)	6.15 (3.8–12.8)	6.8 (4.3–13.4)	6.15 (2.7–1.4)	0.378	6.1 (4.3–13)	7.25 (3.3–14.9)	0.902
Monocytes (×10^9^/L), median (IQR)	0.56 (0.33–0.84)	0.65 (0.38–0.83)	0.44 (0.28–0.87)	0.397	0.54 (0.36–0.82)	0.56 (0.29–0.85)	0.813
Blasts PB (%), median (IQR)	0 (0–1)	0 (0–1)	1 (0–2)	0.017	0 (0–1)	0 (0–1)	0.605
Constitutional symptoms, n (%)	36/78 (46)	16/47 (34)	16/24 (66.7)	0.012	12/31 (38.7)	19/30 (63.3)	0.074
LDH available, n (%) Median [U/L] (IQR)	69/78 (88) 525 (347–700)	41/47 (87) 457 (329–606)	23/24 (96) 609 (463–932)	0.043	30/31 (97) 541 (365–829)	26/30 (87) 541 (321–686)	0.730
CRP available, n (%) Median [mg/L] (IQR)	71/78 (91) 5 (2–12)	47/47 (100) 3 (1–5)	24/24 (100) 21 (11–35)		28/31 (90) 4 (1.25–6)	29/30 (97) 10 (3.5–24.5)	0.005
Albumin available, n (%) Median [g/L] (IQR)	61/78 (78) 40 (37–43)	36/47 (76) 42 (39–43)	21/24 (88) 37 (35–38)	<0.001	31/31 43 (42–44)	30/30 37 (35–38)	
CAR available, n (%) Median (IQR)	57/78 (73) 0.128 (0.051–0.374)	36/57 (63) 0.073 (0.0263–0.125)	21/24 (87) 0.579 (0.315–0.808)	<0.001	28/31 (90) 0.093 (0.029–0.142)	29/30 (97) 0.263 (0.094–0.727)	0.001
Need of transfusion, n (%)	12/78 (15)	2/47 (4.3)	8/24 (33.3)	0.002	3/31 (9.7)	6/30 (20)	0.301
Platelets < 100 × 10^9^/L, n (%)	5/77 (6.5)	0/47 (0)	4/24 (17)	0.011	1/30 (3)	2/30 (17)	1.00
Splenomegaly (clinically or imaging), n (%)	63/78 (81)	37/47 (79)	20/24 (83)	0.759	25/31 (80.6)	26/30 (86.7)	0.731
BMI, available, n (%) Median (kg/m^2^) (IQR)	72/78 (92) 24.5 (21–28)	42/47 (89) 24.4 (21.1–28.3)	23/24 (96) 26 (22.0–28.2)	0.484	27/31 (87) 26.4 (22.9–29.2)	30/30 22.1 (20.4–26.1)	0.008
Driver Mutations							
JAK2-V617F (n, %)	46/78 (59)						
CALR (n, %)	16/78, (20.5)						
MPL (n, %)	3/78, (3.8)						
Triple negative (n, %)	5/78, (6.4)						
Unknown * (n, %)	8/78, (10.3)						

IQR, interquartile range; CAR, CRP/albumin ratio; BMI, body mass index. * cases diagnosed between 2000 and 2009 and no DNA available for retrospective analyses.

**Table 2:** Uni- and bivariate Cox regression models including MIPSS70dich, albumin, CRP, CRP/albumin ratio [CAR] and Glasgow Prognostic Score [GPS] for all cases including primary and secondary myelofibrosis.

	Univariate				Bivariate			
	n	HR	95% CI	*p*	n	HR	95% CI	*p*
MIPSS70dich	59	4.90	1.99–12.0	0.001	56	3.45	1.28–9.32	0.0148
CRP > 8 mg/L	71	3.85	1.85–8.0	<0.001		2.50	1.13–5.52	0.0236
MIPSS70dich					50	8.65	2.87–26.07	<0.001
Albumin < 40 g/L	61	2.49	1.13–5.49	0.024		5.49	1.89–15.96	0.0018
MIPSS70dich					47	4.86	1.99–11.88	0.0005
CAR > 0.204	57	1.84	1.01–3.34	0.046		1.37	0.66–2.84	0.4026
MIPSS70dich					47	5.98	1.84–19.46	0.0030
CAR > 0.374	57	4.25	1.75–10.32	0.001		3.53	1.36–9.17	0.0095
MIPSS70dich					47	6.35	1.95–20.73	0.0022
GPS > 0	57	5.38	2.17–13.37	<0.001		4.63	1.76–12.1	0.0019

**Table 3:** Uni- and bivariate Cox regression models including MIPSS70dich, albumin, CRP, CRP/albumin ratio [CAR] and Glasgow Prognostic Score [GPS] for primary myelofibrosis only.

	Univariate				Bivariate			
	n	HR	95% CI	*p*	n	HR	95% CI	*p*
MIPSS70dich	45	6.26	2.19–17.89	0.0006	42	4.21	1.40–12.65	0.0104
CRP > 8 mg/L	52	3.86	1.52–9.76	0.0044		2.12	0.77–5.87	0.146
MIPSS70dich					37	9.92	2.46–40.0	0.0013
Albumin < 40 g/L	48	2.12	0.91–4.92	0.0823		4.89	1.15–20.78	0.0317
MIPSS70dich					35	10.15	2.61–39.44	0.0008
CAR > 0.204	44	4.06	1.41–11.66	0.0093		2.71	0.85–8.64	0.0923
MIPSS70dich					35	8.33	2.09–33.18	0.0026
CAR > 0.374	44	3.88	1.39–10.80	0.0094		3.38	1.09–10.50	0.0353
MIPSS70dich					35	9.83	2.22–43.60	0.0026
GPS > 0	44	4.60	1.61–13.18	0.0044		4.32	1.33–14.02	0.0148

## Data Availability

The data presented in this study are available on request from the corresponding author.

## Supplementary Materials

The following supporting information can be downloaded at: https://www.mdpi.com/article/10.3390/cancers15051479/s1, Figure S1: Overall survival of MF patients from Cohort A according to MIPSS70; Figure S2: Probability of death according to albumin concentration as a continuous variable; Figure S3: Elevated CRP-levels in MPN and its association with MPN-subtypes and disease progression; Figure S4: Reduced albumin levels in MPN and its association with MPN-subtypes and disease progression; Figure S5: Correlation plots of inflammatory cytokine panel and CRP levels in MPN patients; Figure S6: Correlation plots of inflammatory cytokine panel and CRP levels in MPN patients; Figure S7: Correlation plots of inflammatory cytokine panel and albumin levels in MPN patients; Figure S8: Correlation plots of inflammatory cytokine panel and CRP levels in MPN patients; Table S1: Patient characteristics of Cohort B (used for cytokine assessment; as shown in Figures S2–S8); Table S2: Patient characteristics of patients with MF from Cohort A including PMF (56/78) and MF post ET/PV (22/78) according to levels of CRP and albumin as used in the Glagow prognostic score; Table S3: Levels of albumin, C-reactive protein (CRP) and CRP/albumin ratio (CAR) according to molecular characteristics in patients with MF from Cohort A.

## References

[1] Passamonti F., Mora B. (2022). Myelofibrosis. Blood.

[2] Gangat N., Tefferi A. (2020). Myelofibrosis Biology and Contemporary Management. Br. J. Haematol..

[3] Passamonti F., Cervantes F., Vannucchi A.M., Morra E., Rumi E., Pereira A., Guglielmelli P., Pungolino E., Caramella M., Maffioli M. (2010). A Dynamic Prognostic Model to Predict Survival in Primary Myelofibrosis: A Study by the IWG-MRT (International Working Group for Myeloproliferative Neoplasms Research and Treatment). Blood.

[4] Grinfeld J. (2020). Prognostic Models in the Myeloproliferative Neoplasms. Blood Rev..

[5] Barbui T., Carobbio A., Finazzi G., Guglielmelli P., Salmoiraghi S., Rosti V., Rambaldi A., Vannucchi A.M., Barosi G. (2013). Elevated C-Reactive Protein Is Associated with Shortened Leukemia-Free Survival in Patients with Myelofibrosis. Leukemia.

[6] Veletic I., Manshouri T., Newberry K.J., Garnett J., Verstovsek S., Estrov Z. (2019). Pentraxin-3 Plasma Levels Correlate with Tumour Burden and Overall Survival in Patients with Primary Myelofibrosis. Br. J. Haematol..

[7] Kuykendall A.T., Talati C., Sallman D.A., Sweet K.L., Padron E., Lancet J.E., List A.F., Zuckerman K.S., Komrokji R.S. (2017). Serum albumin is a strong predictor of survival in myelofibrosis, independent of ipss, dipss, and dipss+ scores. Haematologica.

[8] Lucijanic M., Veletic I., Rahelic D., Pejsa V., Cicic D., Skelin M., Livun A., Tupek K.M., Stoos-Veic T., Lucijanic T. (2018). Assessing Serum Albumin Concentration, Lymphocyte Count and Prognostic Nutritional Index Might Improve Prognostication in Patients with Myelofibrosis. Wien. Klin. Wochenschr..

[9] Tefferi A., Nicolosi M., Penna D., Mudireddy M., Szuber N., Lasho T.L., Hanson C.A., Ketterling R.P., Gangat N., Pardanani A.D. (2018). Development of a Prognostically Relevant Cachexia Index in Primary Myelofibrosis Using Serum Albumin and Cholesterol Levels. Blood Adv..

[10] Lucijanic M., Galusic D., Krecak I., Sedinic M., Soric E., Holik H., Perisa V., Moric Peric M., Zekanovic I., Stoos-Veic T. (2020). C Reactive Protein to Albumin Ratio as Prognostic Marker in Primary and Secondary Myelofibrosis. Leuk. Lymphoma.

[11] Lucijanic M., Cicic D., Stoos-Veic T., Pejsa V., Rahelic D., Lucijanic T., Vasilj T., Ivic M., Sedinic M., Kusec R. (2018). Combining Information on C Reactive Protein and Serum Albumin into the Glasgow Prognostic Score Strongly Discriminates Survival of Myelofibrosis Patients. Blood Cells. Mol. Dis..

[12] Guglielmelli P., Lasho T.L., Rotunno G., Mudireddy M., Mannarelli C., Nicolosi M., Pacilli A., Pardanani A., Rumi E., Rosti V. (2018). MIPSS70: Mutation-Enhanced International Prognostic Score System for Transplantation-Age Patients with Primary Myelofibrosis. J. Clin. Oncol. Off. J. Am. Soc. Clin. Oncol..

[13] Arber D.A., Orazi A., Hasserjian R., Thiele J., Borowitz M.J., Le Beau M.M., Bloomfield C.D., Cazzola M., Vardiman J.W. (2016). The 2016 Revision to the World Health Organization Classification of Myeloid Neoplasms and Acute Leukemia. Blood.

[14] Forrest L.M., McMillan D.C., McArdle C.S., Angerson W.J., Dunlop D.J. (2003). Evaluation of Cumulative Prognostic Scores Based on the Systemic Inflammatory Response in Patients with Inoperable Non-Small-Cell Lung Cancer. Br. J. Cancer.

[15] Koschmieder S., Chatain N. (2020). Role of Inflammation in the Biology of Myeloproliferative Neoplasms. Blood Rev..

[16] Rai S., Grockowiak E., Hansen N., Luque Paz D., Stoll C.B., Hao-Shen H., Mild-Schneider G., Dirnhofer S., Farady C.J., Méndez-Ferrer S. (2022). Inhibition of Interleukin-1β Reduces Myelofibrosis and Osteosclerosis in Mice with JAK2-V617F Driven Myeloproliferative Neoplasm. Nat. Commun..

[17] Rahman M.F.-U., Yang Y., Le B.T., Dutta A., Posyniak J., Faughnan P., Sayem M.A., Aguilera N.S., Mohi G. (2022). Interleukin-1 Contributes to Clonal Expansion and Progression of Bone Marrow Fibrosis in JAK2V617F-Induced Myeloproliferative Neoplasm. Nat. Commun..

[18] Patel H.J., Patel B.M. (2017). TNF-α and Cancer Cachexia: Molecular Insights and Clinical Implications. Life Sci..

[19] Senjo H., Onozawa M., Hidaka D., Yokoyama S., Yamamoto S., Tsutsumi Y., Haseyama Y., Nagashima T., Mori A., Ota S. (2022). High CRP-Albumin Ratio Predicts Poor Prognosis in Transplant Ineligible Elderly Patients with Newly Diagnosed Acute Myeloid Leukemia. Sci. Rep..

[20] De Simone G., di Masi A., Ascenzi P. (2021). Serum Albumin: A Multifaced Enzyme. Int. J. Mol. Sci..

[21] Roche M., Rondeau P., Singh N.R., Tarnus E., Bourdon E. (2008). The Antioxidant Properties of Serum Albumin. FEBS Lett..

[22] Allegra A., Pioggia G., Tonacci A., Casciaro M., Musolino C., Gangemi S. (2020). Synergic Crosstalk between Inflammation, Oxidative Stress, and Genomic Alterations in BCR-ABL-Negative Myeloproliferative Neoplasm. Antioxidants.

[23] Arques S. (2020). Serum Albumin and Cardiovascular Disease: State-of-the-Art Review. Ann. Cardiol. Angeiol. (Paris).

[24] Kaptoge S., Di Angelantonio E., Lowe G., Pepys M.B., Thompson S.G., Collins R., Danesh J., Emerging Risk Factors Collaboration (2010). C-Reactive Protein Concentration and Risk of Coronary Heart Disease, Stroke, and Mortality: An Individual Participant Meta-Analysis. Lancet Lond. Engl..

[25] Mesa R.A., Verstovsek S., Gupta V., Mascarenhas J.O., Atallah E., Burn T., Sun W., Sandor V., Gotlib J. (2015). Effects of Ruxolitinib Treatment on Metabolic and Nutritional Parameters in Patients with Myelofibrosis From COMFORT-I. Clin. Lymphoma Myeloma Leuk..

[26] Khosravi N., Stoner L., Farajivafa V., Hanson E.D. (2019). Exercise Training, Circulating Cytokine Levels and Immune Function in Cancer Survivors: A Meta-Analysis. Brain. Behav. Immun..

[27] Caldo-Silva A., Furtado G.E., Chupel M.U., Bachi A.L.L., de Barros M.P., Neves R., Marzetti E., Massart A., Teixeira A.M. (2021). Effect of Training-Detraining Phases of Multicomponent Exercises and BCAA Supplementation on Inflammatory Markers and Albumin Levels in Frail Older Persons. Nutrients.

[28] Mendez L.F., Nguyen H., Nguyen J., Himstead A., Lemm M.R., Heide E.S., Scherber R.M., Choudhry A., McKinney C.O., Mesa R.A. (2019). The Nutrient Trial (NUTRitional Intervention among MyEloproliferative Neoplasms): Feasibility Phase. Blood.

[29] Tefferi A., Saeed L., Hanson C.A., Ketterling R.P., Pardanani A., Gangat N. (2017). Application of Current Prognostic Models for Primary Myelofibrosis in the Setting of Post-Polycythemia Vera or Post-Essential Thrombocythemia Myelofibrosis. Leukemia.

[30] Masarova L., Verstovsek S. (2019). The Evolving Understanding of Prognosis in Post-Essential Thrombocythemia Myelofibrosis and Post-Polycythemia Vera Myelofibrosis vs Primary Myelofibrosis. Clin. Adv. Hematol. Oncol. HO.

[31] Passamonti F., Giorgino T., Mora B., Guglielmelli P., Rumi E., Maffioli M., Rambaldi A., Caramella M., Komrokji R., Gotlib J. (2017). A Clinical-Molecular Prognostic Model to Predict Survival in Patients with Post Polycythemia Vera and Post Essential Thrombocythemia Myelofibrosis. Leukemia.

[32] Vannucchi A.M., Guglielmelli P. (2022). Molecular Prognostication in Ph-Negative MPNs in 2022. Hematology.

[33] Tefferi A., Guglielmelli P., Lasho T.L., Gangat N., Ketterling R.P., Pardanani A., Vannucchi A.M. (2018). MIPSS70+ Version 2.0: Mutation and Karyotype-Enhanced International Prognostic Scoring System for Primary Myelofibrosis. J. Clin. Oncol. Off. J. Am. Soc. Clin. Oncol..

