# Supplementary material for: CRP/Albumin Ratio and Glasgow Prognostic Score Provide Prognostic Information in Myelofibrosis Independently of MIPSS70—A Retrospective Study
Source: Cancers (Basel). 2023 Feb 25;15(5):1479. doi: 10.3390/cancers15051479 (PMC10000567; doi:10.3390/cancers15051479)
Supplement: Supplementary file 1 [file cancers-15-01479-s001.zip › cancers-2186299-supplementary.pdf]

Table S1. Patient characteristics of Cohort B (used for cytokine assessment; as shown in Supplementary Figures 2-8).

Diagnosis	Gender	# of Patients (%)	Median Age at sampling (years)	Driver Mutation	Treatment (# of Patients)	CRP levels (mg/dL) IQR / Median	Albumin levels (g/L) IQR / Median
ET	Male	13 (16.25)	67 (28 - 81)	JAK2-V617F (n=13)	n/a	1.02 / 0.75	31.63 / 27.20
ET	Female	5 (6.25)	51 (42 - 79)	JAK2-V617F (n=5)	n/a	1.06 / 1.04	31.63 / 30.67
PV	Male	13 (16.25)	65 (49 - 87)	JAK2-V617F (n=13)	n/a	0.92 / 0.83	23.54 / 20.33
PV	Female	5 (6.25)	70 (40 - 79)	JAK2-V617F (n=5)	n/a	0.79 / 0.75	26.31 / 17.11
PMF	Male	18 (22.5)	60.5 (56 - 82)	JAK2-V617F / (n=18) CALR del52 (n=3)	Ruxolitinib (2) Momelotinib (2) Hydroxyurea (1) Anagrelide (2)	1.35 / 1.23	27.21 / 26.31
PMF	Female	10 (12.5)	61.5 (50 - 78)	JAK2-V617F (n=18) CALR del52 (n=3)	Ruxolitinib (3) Momelotinib (2) Hydroxyurea (3) Anagrelide (2)	1.16 / 0.96	27.11 / 23.21

Table S2. Patient characteristics of patients with MF from cohort A including PMF (56/78) and MF post ET/PV (22/78) according to levels of CRP and albumin as used in the Glasgow Prognostic Score.

	**Whole population**	**CRP ≤10mg/l**	**CRP >10 mg/l**	**p**	**Albumin ≥35 g/l**	**Albumin <35g/l**	**p**
n	78	52	19		55	6	
Age<br>[years], median, (IQR)	72<br>(60-78)	70<br>(60-77)	77<br>(61-80)	0.137	73<br>(62-78)	75<br>(66-77)	0.915
Female<br>n, (%)	37<br>(46.2)	25/52<br>(48)	9/19<br>(47)	1	27/55<br>(49)	3/6<br>(50)	1
Bone Marrow fibrosis grade 2 n (%)	54/78<br>(70)	40/52<br>(77)	11/19<br>(57)		42/55<br>(76)	3/6<br>(50)	
Bone Marrow fibrosis grade 3 (n, %)	24/78<br>(30)	12/52<br>(23)	8/19<br>(42)	0.141	13/55<br>(24)	3/6<br>(50)	0.179
Hemoglobin [g/l],<br>median (IQR)	107<br>(88-122)	115<br>(102-130)	82<br>(75-95)	**<0.001**	112<br>(88-124)	93<br>(75-96)	**0.022**
Platelet count ($\times 10^9$/l),<br>median (IQR)	410<br>(197-663)	535<br>(344-772)	218<br>(106-334)	**<0.001**	484<br>(227-696)	288<br>(89-719)	0.249
Leukocytes ($\times 10^9$/l),<br>median (IQR)	8.9<br>(6.0-15.6)	9.5<br>(6.8-15.9)	8.9<br>(5.2-20.8)	0.599	9.4<br>(6.7-16.4)	10.9<br>(4.6-17.2)	0.714
Neutrophils ($\times 10^9$/l),<br>median (IQR)	6.2<br>(3.8-12.8)	6.7<br>(4.3-13.3)	6.1<br>(2.6-15.2)	0.443	6.5<br>(4.3-13.4)	8.4<br>(2.4-13.4)	0.823
Monocytes ($\times 10^9$/l),<br>median (IQR)	0.57<br>(0.33-0.84)	0.67<br>(0.38-0.84)	0.42<br>(0.28-0.83)	0.132	0.57<br>(0.35-0.83)	0.36<br>(0.1-1.1)	0.303
Blasts PB (%),<br>Median, IQR	0<br>(0-1)	0<br>(0-1)	1<br>(0-2)	0.077	0<br>(0-1)	0.5<br>(0-1)	0.859
Constitutional Symptoms n (%)	36/78<br>(46)	19/52<br>(37)	13/19<br>(68)	**0.030**	26/55<br>(47)	5/6<br>(83)	0.195
LDH available, n (%)<br>median [U/l]<br>(IQR)	69/78 (88)<br>525<br>(347-700)	45/52 (86)<br>492<br>(329-659)	19/19 (100)<br>564<br>(462-848)	**0.174**	50/55 (91)<br>551<br>(331-730)	6/6 (100)<br>506<br>(277-657)	0.614
CRP available n (%)<br>median [mg/l],<br>(IQR)	71/78 (91)<br>5<br>(2-12)	52/52 (100)<br>3<br>(1-5)	19/19 (100)<br>28<br>(14-40)	**<0.001**	51/55 (93)<br>5<br>(2-10)	6/6 (100)<br>17<br>(10-105)	**0.009**
Albumin available, n (%)<br>median [g/l]<br>(IQR)	61/78 (78)<br>40<br>(37-42.7)	40/52 (77)<br>42.1<br>(38.1-43)	17/19 (89)<br>37<br>(32-38.2)	**<0.001**	55/55 (100)<br>41<br>(37.9-42.8)	6/6 (100)<br>29.1<br>(26.4-30.9)	<0.001
CAR available, n (%)<br>Median<br>(IQR)	57/78 (73)<br>0.128<br>(0.051-0.374)	40/52 (77)<br>0.088<br>(0.028-0.138)	17/19 (89)<br>0.714<br>(0.451-1.152)	**<0.001**	51/55 (93)<br>0.116<br>(0.047-0.263)	6/6 (100)<br>0.628<br>(0.306-3.575)	**0.002**
Need of transfusion, n (%)	12/78<br>(15)	2/52<br>(4)	8/19<br>(42)	**<0.001**	6/55<br>(11)	3/6<br>(50)	**0.037**
Platelets <100×10^9/l, n (%)	5/77<br>(6.5)	0/52<br>(0)	4/19<br>(21)	**0.004**	3/54<br>(6)	1/6<br>(17)	0.351
Splenomegaly (clinically or imaging), n (%)	63/78<br>(81)	41/52<br>(79)	16/19<br>(84)	0.745	46/55<br>(84)	5/6<br>(83)	1.0
BMI, available n (%)<br>Median (kg/m^2)<br>(IQR)	72/78 (92)<br>24.5<br>(21.2-28)	47/52 (90)<br>25.6<br>(21.5-28.4)	18/19 (95)<br>24.5<br>(21.4-26.8)	0.649	51/55 (93)<br>24.5<br>(21.6-28.4)	6/6 (100)<br>20.5<br>(19.4-25.9)	0.062

IQR, Interquartile Range, CAR, CRP/Albumin-Ratio, BMI, Body Mass Index

Table S3. Levels of albumin, C-reactive protein (CRP) and CRP/albumin-Ratio (CAR) according to molecular characteristics in patients with myelofibrosis from cohort A:.

	**Albumin [g/l] Median (IQR)**	**p**	**CRP [mg/l] Median, IQR**	**p**	**CAR Median, IQR**	**p**
			**Driver Mutation**			
JAK2-V617F (n=46)	39 (37-42)	0.302	5 (3-12)	0.131	0.128 (0.061-0.31)	0.451
CALR (n=16)	42 (35-44)		2 (1-11)		0.077 (0.025-0.482)	
MPL (n=4)	44 (41- .)		5 (1-.)		0.0228 (0.114- .)	
Triple negative (n=5)	36 (29-37)		20 (6-117)		0.733 (0.242-4.477)	
			**Mutational Burden**			
JAK2-V617F <50%	39 (37-43)	0.158	4.5 (1-13.25)	0.071	0.095 (0.025-0.251)	**0.035**
JAK2-V617F <50%	38 (33-41)		7.5 (4.8-18)		0.243 (0.113-0.684)	
			**High-Risk Mutations according to MIPSS70 (ASXL1, EZH2, SRSF2, IDH1/2)**			
absent	39 (35-43)		5 (1-11-25)	0.157	0.115 (0.278-0.298)	
present	38 (37.8-42)	0.801	7 (3-28)		0.579 (0.095-0.751)	0.051

Supplementary Figures

Figure Legends

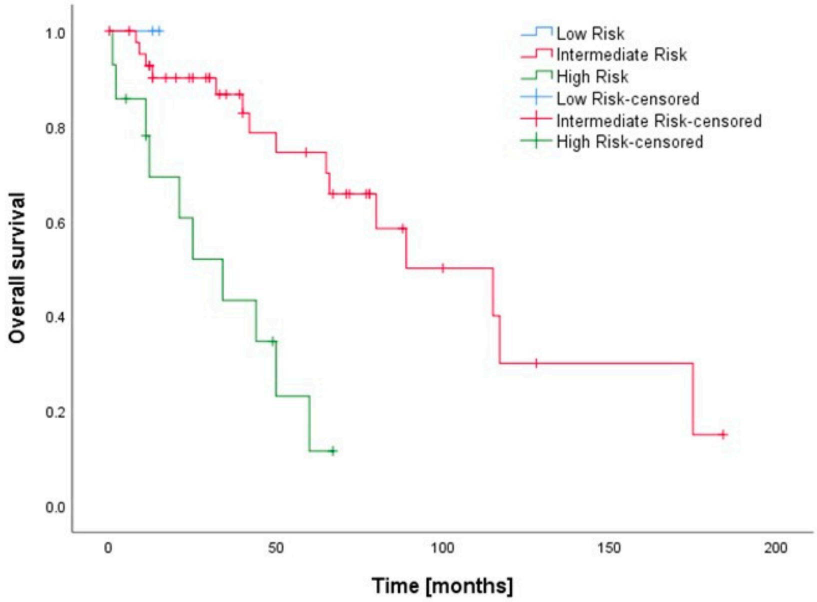

MIPSS70 group	n	Median OS [months]	95% CI	P
low	2	n.a.		<0.001
Intermediate	43	115	65-175	
high	14	25	11-50	

Figure S1. Overall survival of MF patients from Cohort A according to MIPSS70.

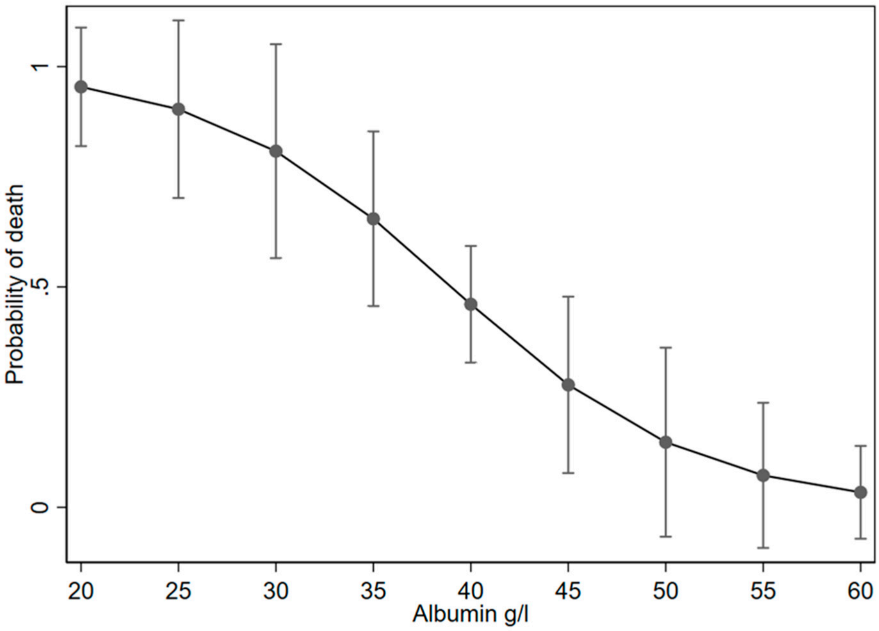

Figure S2. Probability of death according to albumin concentration as a continuous variable. Association of albumin concentration and probability of deaths in an unadjusted logistic regression model for patients of cohort A

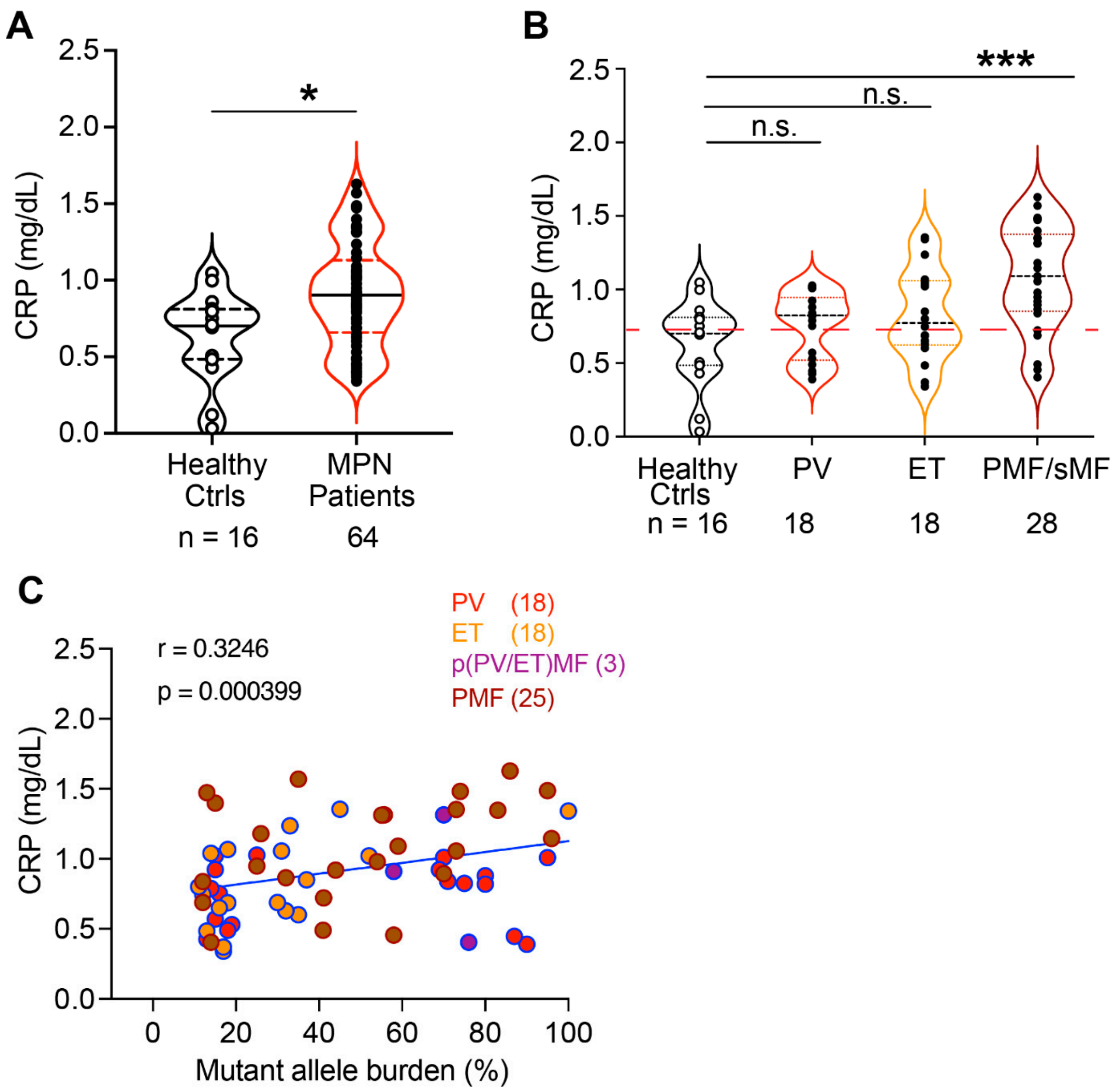

Figure S3. Elevated CRP levels in MPN and its association with MPN-subtypes and disease progression. A). CRP levels in MPN patients (n=64) and healthy donors (n=16). (B). Patients shown in panel A wwere used to assess CRP levels in MPN subtypes. C). Correlation (r, Pearson correlation) and significance (2-tailed Student t-test) between JAK2-V617F or CALR-del52 mutant allele burden measured in PB granulocytes and CRP levels in plasma. All data are means+/- standard error of the mean (SEM); *n.s.*, not significant; **P < 0.01; ***P < 0.001.

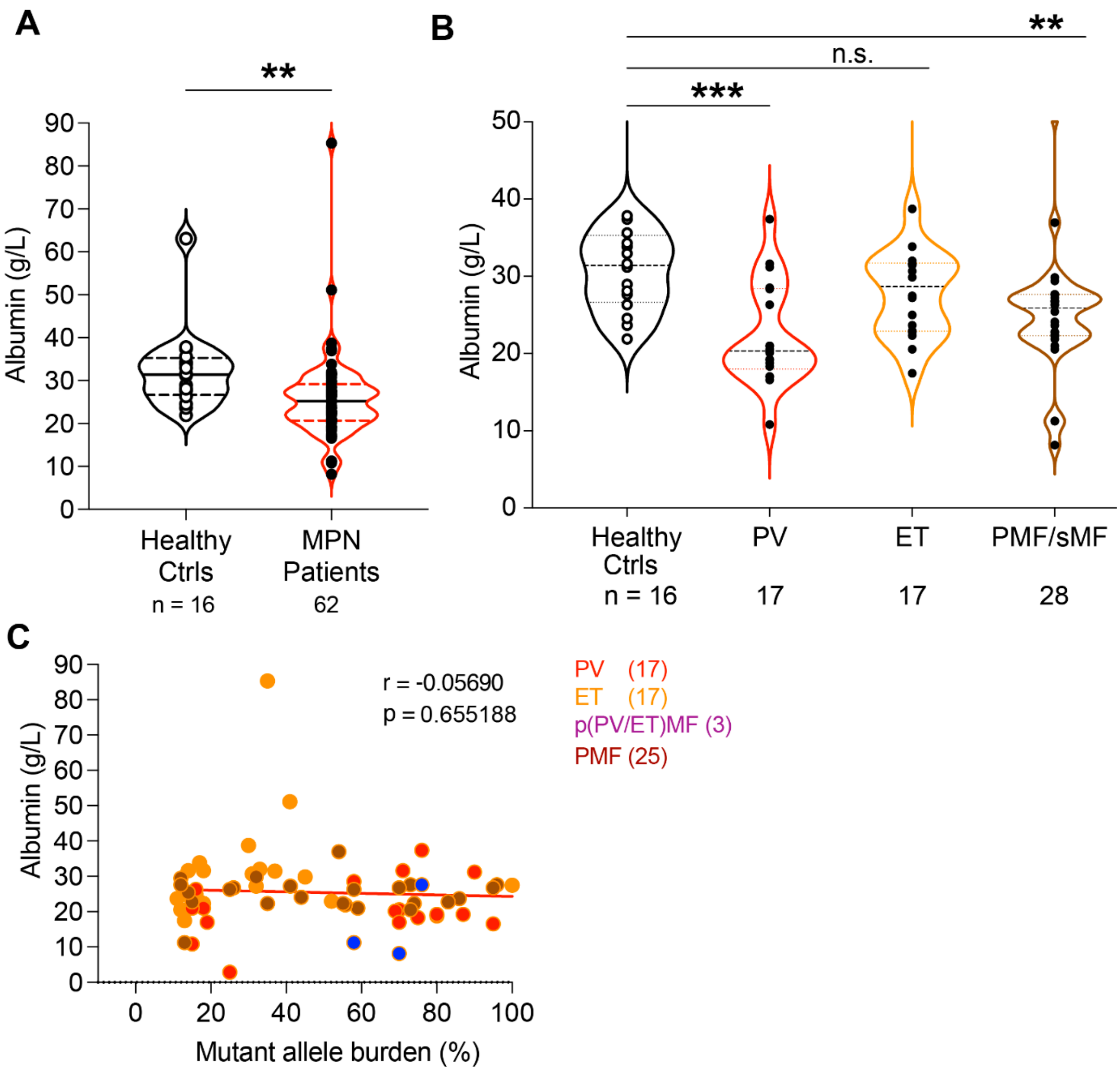

Figure S4. Reduced albumin levels in MPN and its association with MPN-subtypes and disease progression. A). Albumin levels in MPN patients (n=62) and healthy donors (n=16). (B). Patients shown in panel A were used to assess albumin levels in MPN subtypes. C). Correlation (r, Pearson correlation) and significance (2-tailed Student t-test) between JAK2-V617F or CALR-del52 mutant allele burden measured in PB granulocytes and albumin levels in plasma. All data are means+/- standard error of the mean (SEM); *n.s.*, not significant; **P < 0.01; ***P < 0.001.

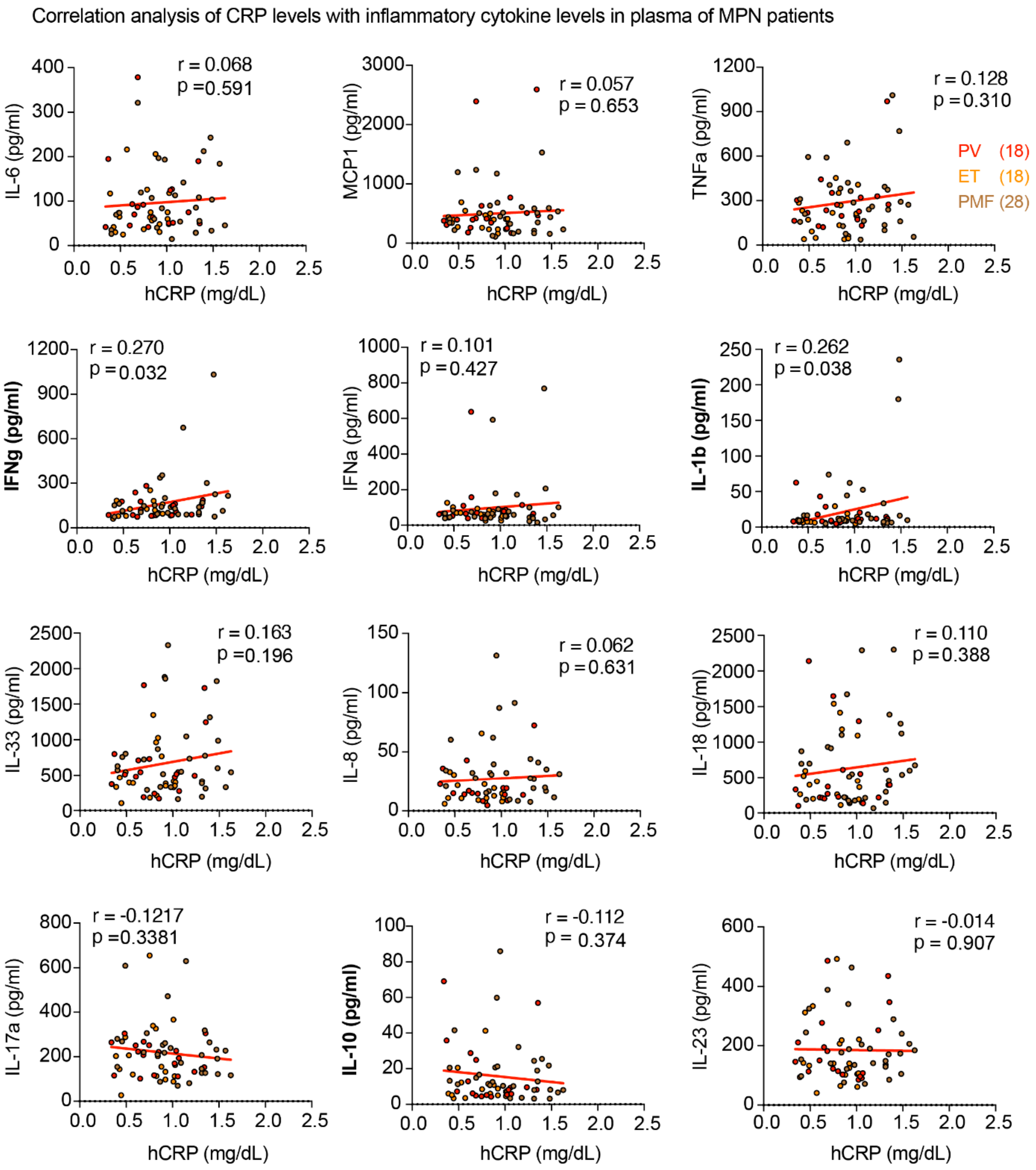

Figure S5. Correlation plots of inflammatory cytokine panel and CRP levels in MPN patients. Correlation (r, Pearson correlation) and significance (2-tailed Student t-test) between CRP and indicated inflammatory cytokine levels in plasma of MPN patients.

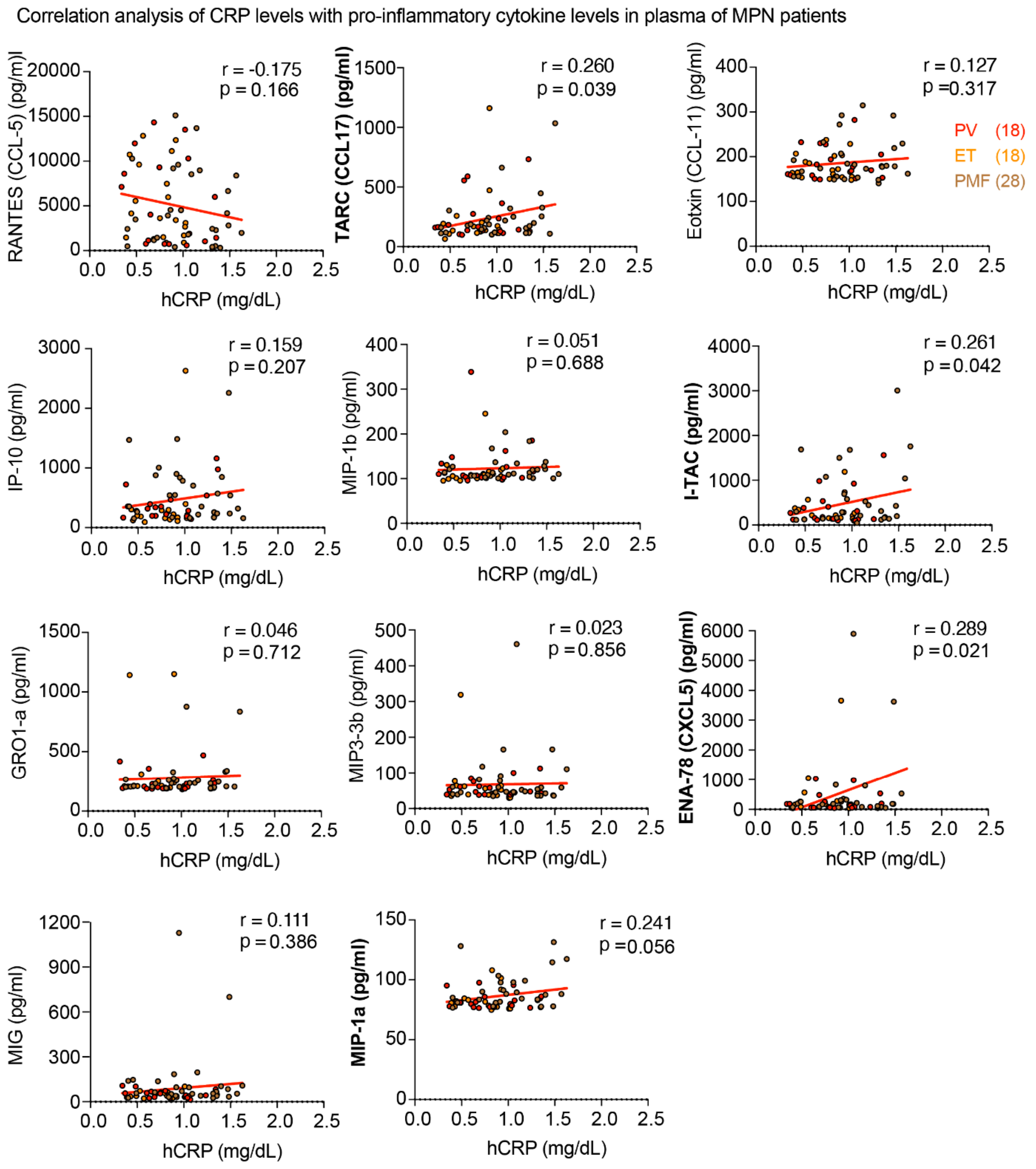

Figure S6. Correlation plots of pro-inflammatory cytokine panel and CRP levels in MPN patients. Correlation (r, Pearson correlation) and significance (2-tailed Student t-test) between CRP and indicated pro-inflammatory cytokine levels in plasma of MPN patients.

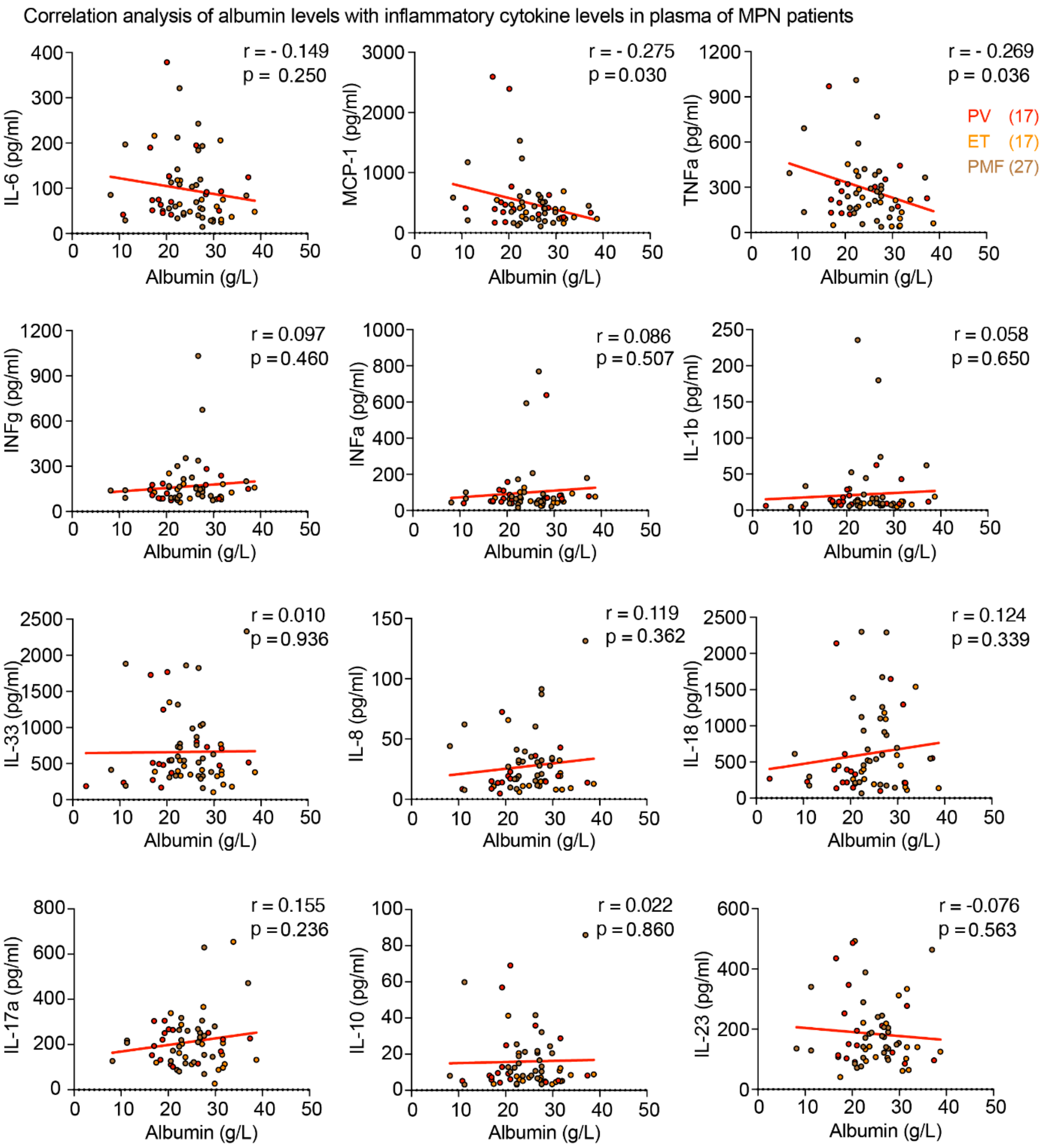

Figure S7. Correlation plots of inflammatory cytokine panel and albumin levels in MPN patients. Correlation (r, Pearson correlation) and significance (2-tailed Student t-test) between albumin and indicated inflammatory cytokine levels in plasma of MPN patients.

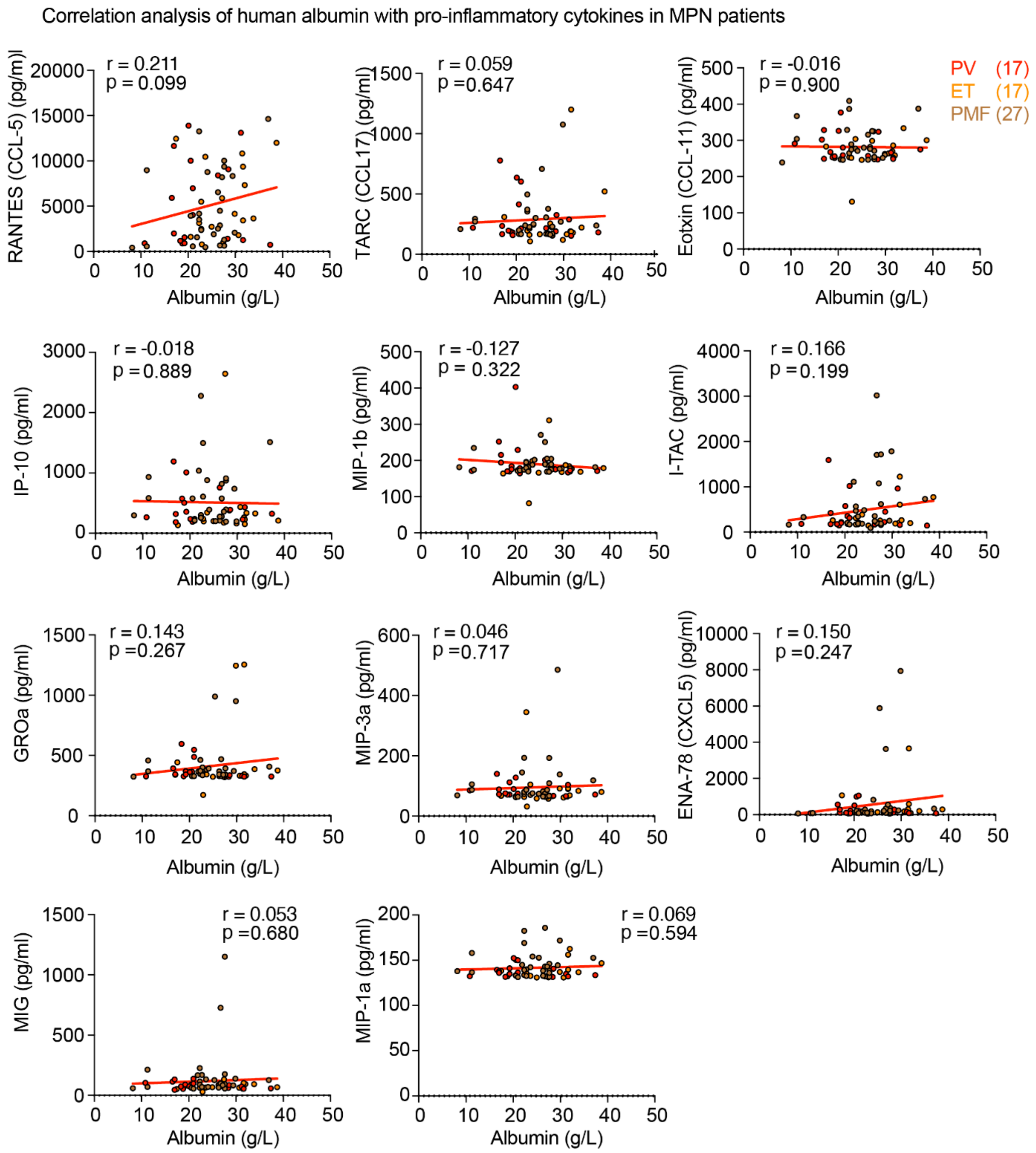

Figure S8. Correlation plots of pro-inflammatory cytokine panel and albumin levels in MPN patients. Correlation (r, Pearson correlation) and significance (2-tailed Student t-test) between albumin and indicated pro-inflammatory cytokine levels in plasma of MPN patients.

Supplementary methods

Next generation sequencing

DNA was isolated from bone marrow aspirate or biopsy samples by automated DNA purification on a Maxwell® RSC instrument (Promega, Fitchburg, WI, USA). DNA concentration was measured with the Qubit™ dsDNA BR (Broad Range) Assay Kit (Thermo Fisher Scientific, San Francisco, CA, USA). For NGS-based mutation analysis, the DNA panel of the Oncomine™ Myeloid Research Assay (OMRA; Thermo Fisher Scientific,) was used that targets all exonic regions of 17 genes and exonic hotspots of 23 genes (526 amplicons). OMRA libraries were prepared manually according to the manufacturer's instructions (Thermo Fisher Scientific). Briefly, barcoded libraries were generated from 30 ng of sample genomic DNA. Targets were amplified using multiplex PCR. Amplification products were partially digested with Fupa enzyme, followed by ligation of unique barcode adapters for each library. The barcoded libraries were quantified by qPCR using the KAPA Library Quantification Kit (Roche) and diluted to 40 pmol/L. Templating and clonal amplification of sample libraries onto Ion Sphere Particles by emulsion PCR were performed with the Ion Chef™ instrument (Thermo Fisher Scientific). Enriched Ion Sphere Particles were loaded onto 510, 520, or 530 chips using an Ion 510™ & Ion 520™, or Ion 530™ Kit. A total of 3, 4 and 20 samples per 510, 520 and 530 chip, respectively, were run in the automated templating preparation on the Ion Chef™. Sequencing was performed on an Ion GeneStudio™ S5 instrument (Thermo Fisher Scientific). The Torrent Suite software version 5.10.0 (Thermo Fisher Scientific) was used for sequence alignment to reference genome hg19 and base calling. Coverage maps were generated using the Coverage Analysis plugin version 5.16.0 (Thermo Fisher Scientific). Variant identification and annotation were performed using Ion Reporter™ (IR) software version 5.16 (Thermo Fisher Scientific) using the default analysis parameter settings of the Oncomine Myeloid Research workflow. In these settings, the minimum coverage requirement for the analysis is 20 for both SNVs and indels, and 15 for hotspots; the minimum cutoff variant allele fraction (VAF) is 2.5% for both SNVs and indels and 3% for hotspots; and the maximum strand bias tolerance is 0.9 for SNVs, 0.85 for indels, and 0.96 for hotspots.

Quantification of CRP (C-reactive protein) levels in patients' plasma

Peripheral blood was collected in heparin coated tubes and plasma was prepared by centrifugation at 12000 rpm for 10 minutes. Plasma CRP levels were measured by sandwich ELISA (Enzyme-Linked Immunosorbent Assay) kit (Cat# KE00037,

Proteintech) according to the manufacturer's protocol. Plasma samples were diluted 1:200 in sample dilution buffer. CRP concentration was quantified with the standard curve and expressed in mg/dL.

Quantification of albumin levels in patients' plasma

Plasma albumin concentrations were measured by BCG (Bromocresol Green) colorimetric assay as per the manufacturer's (Cat# MAK124, Sigma Aldrich) instructions. Briefly, BCG reagent was added to the three-fold diluted plasma samples and albumin standards, incubated for 5 minutes at room temperature with 500 rpm in a 96-well plate shaker. The absorbance measured at 600 nm (Tekan analyzer) is directly proportional to the plasma albumin concentration was quantified with the standard curve, albumin levels were expressed in g/L.

Inflammatory and pro-inflammatory cytokine measurement by cytokine arrays

Inflammatory and pro-inflammatory cytokine levels in plasma of MPN patients and healthy donors were measured by flow cytometry-based assays with Legendplex human inflammation panel (13 plex, Cat# 740118, Biolegend) and Legendplex human pro-inflammation panel (13-plex, Cat# 740003, Biolegend), respectively. Samples were analyzed on a BD Fortessa analyzer (BD bioscience) and the data was processed using FACS-Diva software. Mean fluorescence intensity (MFI) of individual cytokines and chemokines were interpolated with the MFI of standards provided in the assay kit.

Statistics

Categorical variables were analysed by frequency tables and compared by the $\chi 2$-test, continuous variables were described by median and Inter Quartile Range (IQR) and compared by the Mann-Whitney-U (comparison of two groups) or the Kruskal–Wallis test (comparison of ≥3 groups), since data did not follow a normal distribution. Correlations between continuous variables were assessed by Spearman-Rho and between categorical variables by Cramér's V. Overall survival was calculated in months from the date of diagnosis to the respective event date, i.e. death or censoring. Patients having received an allogeneic stem cell transplant were censored at the time of transplantation. Kaplan-Meier estimates with log-rank test and Cox proportional hazard regression models with robust standard errors were employed to estimate the

unadjusted and adjusted survivor functions. We reported survivor functions and hazard ratios (HR) with corresponding 95% confidence intervals (95% CI). Furthermore, we used an unadjusted logistic regression model to explore the association between albumin levels and death and reported Odds Ratios (OR) with corresponding 95% CI. All statistical tests were two-sided and a p value < 0.05 was considered statistically significant. All analyses were performed with IBM SPSS Statistics for Windows, (Version 25.0. IBM Corp, Armonk, NY, USA) or with Stata (Version 17.0, StataCorp, College Station, TX, USA).

The significance for CRP and albumin levels between healthy donors and MPN patients was determined with unpaired, non-parametric, Kolmogorov-Smirnov test. Pearson correlation (r) with simple linear regression (95% confidence interval) and two-tailed analysis was used for the correlation analysis of CRP and albumin with variant alle frequency (VAF) or cytokine levels (Graphpad Prism V9).
